# American Medical Association

**Published:** 1869-03

**Authors:** James F. Hibberd

**Affiliations:** Richmond, Ind.


### American Medical Association.

*Meeting at New Orleans, Tuesday, May 4th,* 1869.

I am authorized by the Atlantic and Mississippi Steamship Co., of St. Louis, to say, that they will carry Doctors and their Ladies to attend the meeting of the Association, at the following rates, viz.:

| | |
|---|---|
| From St. Louis to New Orleans, each passenger, - - - - | $20 00 |
| " Cairo " " " " - - - - | 18 00 |
| " Memphis " " " " - - - - - | 15 00 |
| Returning, | |
| From New Orleans to Memphis, each passenger, - - - - | $15 00 |
| " " " Cairo, " " - - - - | 18 00 |
| " " " St. Louis, " " - - - - | 20 00 |

The Company start a first class Steamer from St. Louis every forty-eight hours, Sundays included, and the usual time from St. Louis to New Orleans, is about six days, and from Cairo to New Orleans, about four and a half days. Passengers can go on any of their boats at the above rates, which includes meals and state-rooms.

The Steamer which will, however, take down the great body of the Doctors, wishing to travel by the river, will leave St. Louis at 5 o'clock P. M., on Wednesday, the 28th of April; Cairo on Thursday evening, after the arrival of the afternoon train on the Illinois Central Railroad; and Memphis on Friday evening, reaching New Orleans from Monday noon to Tuesday morning.

Parties arriving by railroad, to take this boat, at either St. Louis, Cairo, or Memphis, had better make their calculations to reach the point of embarkation, at least one train in advance of the time of the boat's departure. But, if any one should arrive at Cairo or Memphis too late for this boat. he will find one or more boats passing for New Orleans every day, at ordinary fare.

It was deemed best to make the arrangement for a definite fare each way, so that one can go either down or up, or both, as he may choose, by the river, and know in advance just what he will have to pay.

To avail himself of this boat, one may apply on board, making it known that he is on his way to attend the Association, or, perhaps better, write me a line as early as convenient, stating how many ladies, if any, will accompany him.

Good Steamers, also leave Louisville for New Orleans every two or three days, occupying from six to seven days in the passage down. If a considerable number of Doctors should wish to take passage from Louisville, and would make application in a body to E. T. Sturgeon, Superintendent Louisville & New Orleans Packet Co., at Louisville, or the Captain of a Steamer, starting at the proper time, he would probably give them a liberal reduction from the ordinary fare, which varies from thirty to forty dollars, according to the style and accommodation of the boat.

From Cincinnati no suitable boat can be taken through to New Orleans, but the Cincinnati & Louisville U. S. Mail Line, will take one going to the Association from Cincinnati to Louisville on one of their fine boats, and from thence to New Orleans by rail, for forty dollars, and return him on the same route to Cincinnati free. Two mail boats leave Cincinnati every day at 12 M., and 6 o'clock P. M., except Sundays, one at 12 M. I am not advised as to what arrangements have been made with other railroads.

JAMES F. HIBBERD, M. D.,
Richmond, Ind.

